# An experimental demonstration of irreversible mesoscopic carrier transport phenomena in InGaN quantum wells

**DOI:** 10.1038/s41598-025-20715-1

**Published:** 2025-10-21

**Authors:** Anri Sakurai, Hirokazu Hori, Kazuharu Uchiyama, Akira Ishikawa, Kiyoshi Kobayashi, Katsumi Kishino, Masaru Sakai

**Affiliations:** 1 Department of Science and Advanced Materials, University of Yamanashi, 4‑3‑11 Takeda, Kofu, Yamanashi 400‑8511 Japan; 2 Sophia Nanotechnology Research Center, Sophia University, 7‑1 Kioi‑cho, Chiyoda‑ku, Tokyo 102‑8554 Japan

**Keywords:** Materials science, Nanoscience and technology, Optics and photonics, Physics

## Abstract

Light-induced carrier transport in mesoscopic systems exhibits a complex interplay between classical and quantum phenomena. Through direct spectroscopic measurements of optoelectronic energy transport in semiconductor quantum wells, we reveal the irreversible nature of carrier dynamics in the mesoscopic regime. A 2-probe near-field optical microscopy setup based on multiprobe scanning tunnelling microscopy detected the local excitation and emission at nanoscale resolution. By systematically exchanging the roles of the excitation and detection probes, we demonstrate a clear asymmetry in the spectroscopic response, indicating directional and irreversible transport behaviour. Our proposed approach directly reveals irreversible carrier transport in mesoscopic domains and can probe local excitonic dynamics, opening pathways for designing novel optoelectronic devices with irreversible transport mechanisms.

## Introduction

Mesoscopic phenomena between quantum and classical limits involve various complex interactions that depend on local environmental conditions and observation methods. Such mesoscopic complexity can potentially deliver unprecedented functionality. Remarkable features of coupled electronic–electromagnetic systems and mesoscopic optical systems have been demonstrated in theory^1,2^ but have not been sufficiently verified in experiments. Therefore, experimental techniques are required to evaluate the detailed local states and properties of mesoscopic systems. Direct measurements by our team have clarified that excited carriers transport opto-electronic energy through the semiconductor nanostructure. Using a scanning tunnelling microscope (STM)-assisted multiprobe (M-probe) scanning near-field optical microscope (SNOM) spectroscopy system, we previously observed electromagnetic energy transport in mesoscopic regions^3^. The present paper reports the details of the transport properties observed with our improved M-probe SNOM, revealing that local structural variations or nanometre-scale modifications generate irreversible features in a semiconductor quantum well.

**Fig. 1:**
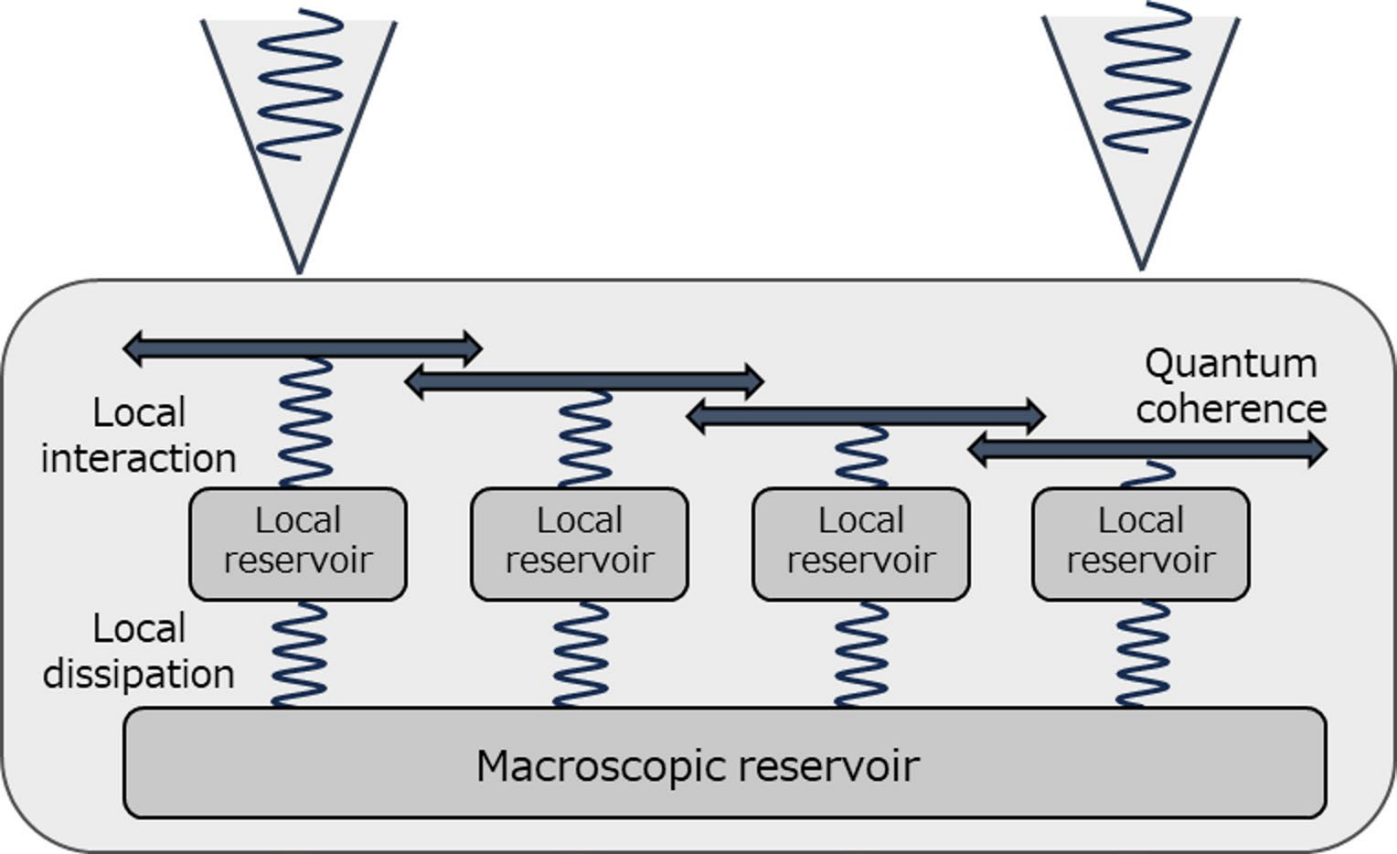
Schematic showing excitation transport in mesoscopic systems under environmental influences. Environmental systems consist of multiple reservoirs on local and macroscopic scales. In mesoscopic systems, where observation processes and other environmental factors can influence the excitation transport, both the classical (non-coherent) and quantum (coherent) dynamics must be considered.

When studying excitation transport processes in mesoscopic systems, we must consider both the classical (non-coherent) and quantum (coherent) dynamics under the influence of the environmental system, including the observation processes^4^. Classical transport refers to the transport of carriers within environmental systems such as potential gradients in the materials. When evaluating quantum behaviour, we must instead consider the intermediate-range correlations between the classical transport paths. As shown in Fig. 1, an environmental system consists of multiple local reservoirs coupled to excitonic carriers through optical phonon resonance or resonant transitions^5–9^. Furthermore, all transport properties between the near-field optical source and sink are governed by a macroscopic reservoir coupled to each local reservoir, each operating as an individual environmental system. As the states of an environmental system cannot be observed, we propose a two-probe measurement approach for predicting and evaluating the influence of the environmental system on carrier transport.

By observing optical near-field effects in the mesoscopic region, SNOM can effectively analyse the behaviour of carriers in quantum structures with local nanometre structural changes^10–13^. Optical near-field effects directly manifest from carrier transport generated in the mesoscopic region. Therefore, SNOM can detect excitonic states associated with carrier-density changes in the sample. When carriers move between the near-field optical source and sink—a process called *optical near-field transport*—local carriers are transported through semiconductor quantum structures and electromagnetic energy is transported over intermediate ranges through optical near-field interactions between carriers. Local spectroscopy of excitonic emission reveals the excitation, transport, and recombination dynamics within a sample, providing information on the initial state, final state, and transport process in the sample. Conventional SNOM is limited to local excitation or detection with a single probe; however, the proposed two-probe method enables carrier transport measurements based on correlation measurements between spatially separated pairs of excitation and detection points^14–17^.

Previously, the transport of near-field optical excitation in the mesoscopic region was directly observed using an STM-assisted M-probe SNOM system. By combining the C-mode and two-probe measurements, we obtained spatially resolved emission spectra and constructed a two-dimensional map of the local potential depths and emission intensities^3^. The carriers diffuse along the gradient of the local potential topography, avoiding high-potential regions and following the paths leading to saddle points. The luminescent intensity also depends on the potential gradient; in particular, radiative recombination tends to enhance along gentle gradients. The integrated intensity maps were then divided into several energy sections, demonstrating that the spectra reflect the recombination behaviour.

Exploiting the advantages of M-probe SNOM, we investigate the irreversibility in mesoscopic transport of excited carriers generated in InGaN/GaN multiple quantum well (MQW) structures with nanoscale structural inhomogeneities. To directly specify the start and end points of the carrier transport, we performed localized photoluminescence (PL) spectroscopy using two spatially separated probes. We also determined the directionality and potential asymmetry in the carrier-transport process by swapping the excitation and detection probes. By adopting this bidirectional approach, we could derive the local state density from the spectral features of the local PL and the behaviour of the photo-excited carriers diffusing across the sample. The results demonstrate clear irreversibility in mesoscopic carrier transport, highlighting the influence of nanoscale environmental interactions. These findings may aid our understanding of carrier dynamics in nanoscale systems and guide suggestions for potential pathways based on irreversible transport properties in novel device applications.

## Sample and experimental setup

The experiments were performed on InGaN/GaN MQW with an emission peak of approximately 2.7 eV. In the quantum structure of InGaN, the excited carriers diffuse through fluctuations in the potential energy field, which is determined by the indium composition^18^. The InGaN MQW used for the measurements comprises five 2-nm active layers with an indium composition of ~ 30%, separated by 5-nm GaN barrier layers. These layers were sequentially deposited on a GaN buffer layer, followed by a 20-nm GaN cap layer. This sample emits blue-green light with high quantum efficiency, even at room temperature, and a 10 nm indium tin oxide layer was sputter-coated onto the sample surface as a conductive film to enable positional control between the sample and probe in the STM method.

**Fig. 2:**
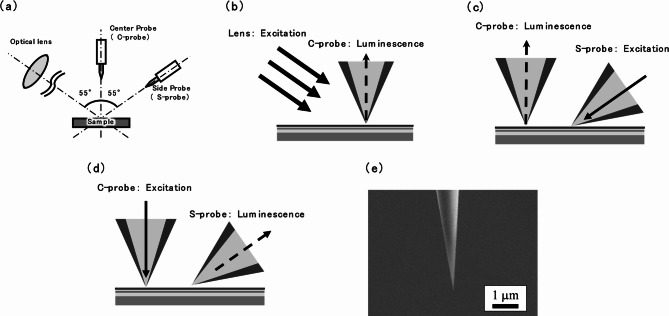
(a) Schematic of the M-probe SNOM. The optical lens and S-probe are positioned at 55° from the C-probe and are independently moveable. (b)–(d) Schematics of the SNOM operations: (b) C-mode operation, (c) normal 2-probe operation with S-probe excitation and C-probe detection, and (d) reversed 2-probe operation with C-probe excitation and S-probe detection. (e) Typical SEM image of the optical fibre probe tip.

The STM-assisted M-probe SNOM consists of an optical system (an excitation source plus a detection system) and an SPM system (UNISOKU USM-1400). The SNOM system also includes a lens for wide-area excitation, a centre probe (C-probe), and a side probe (S-probe) (see Fig. 2a).

To detect signals related to the sample state, the SNOM was operated in two modes: (1) setting the initial state through localized light excitation and carrier generation (local illumination mode; I-mode), and (2) measuring the final state based on carrier detection using a probe (local collection mode; C-mode).

In the C-mode single-probe measurements shown in Fig. 2b, the sample was excited through a classical optical lens (diameter: 10 mm, focal length: 15 mm) mounted on the side and the light emitted from the sample was locally observed with a C-probe. For simplicity, single-probe measurements are hereafter referred to as *C-mode measurements*.

In the 2-probe measurements, an S-probe was employed instead of the optical lens (Fig. 2c). The sample was locally excited by the S-probe and locally observed by a C-probe. In the reversed 2-probe operation, the sample was locally excited by the C-probe and locally observed by the S-probe (Fig. 2d). During S-probe detection, spectroscopic measurements were performed using a single-mode optical fibre, similar to C-probe detection.

The optical fibre probe was formed into a pencil shape using a membrane method with hydrofluoric acid^16,19–21^. Figure 2e shows a scanning electron microscope (SEM) image of a typical optical fibre probe tip. The probe was coated with an approximately 100-nm-thick gold layer using ion beam sputtering. As far as possible, the sample was maintained parallel to the optical aperture created by tapping the probe tip against the sample.

The light source was a frequency-doubled 400-nm titanium sapphire laser (Coherent Mira 900-P; centre wavelength = 800 nm; pulse width = 3 ps, repetition rate = 76 MHz). The light emitted from the light source was focused onto an optical fibre via an optical lens and transmitted to the SNOM system. In addition, the light emitted from the probe-detected sample was measured using a spectrometer (Princeton Instruments SP-2300i; focal length = 30 cm, grating grooves = 600/mm) equipped with a Si-charge-coupled device detector cooled by liquid nitrogen (Roper Scientific Spec-10) via a multimode optical fibre. The measurement system is detailed in Ref. 3.

## Experimental results and discussion

### C-mode measurement

**Fig. 3:**
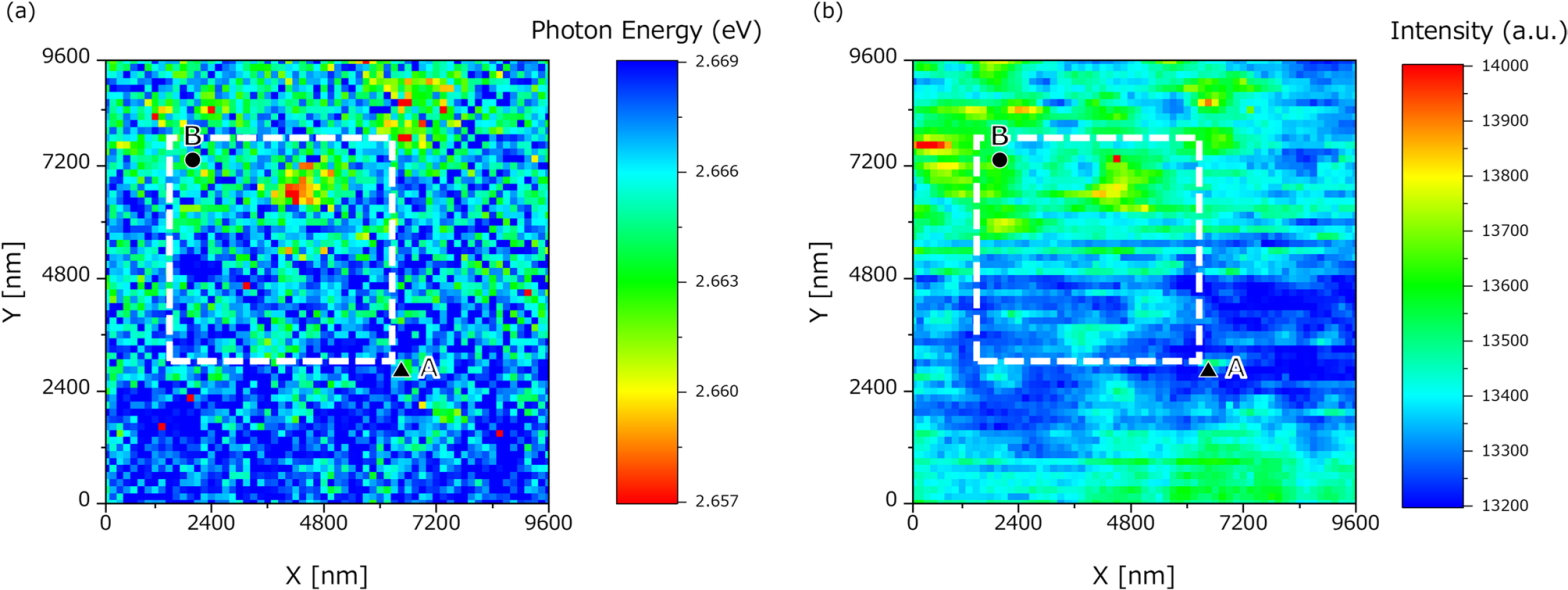
Maps of (a) energy potentials and (b) integral intensities produced in C-mode operation. Two-probe measurements were performed within the square enclosed by the white dashed lines. Excitation and detection were performed at points A (filled upward triangle) and B (filled circle).

The 2-probe correlation measurements were preceded by local PL spectroscopy over a 9.6 μm × 9.6 μm area at 150-nm spatial intervals in C-mode using a C-probe. This and all subsequent experiments were conducted at room temperature. The PL spectrum at each of the 4,096 obtained points was fitted to a Lorentzian function and the peak emission energies in the spectra were compiled into an energy map of the sample (Fig. 3a). In addition, an integrated intensity map across the full spectral range was generated at each observation point (Fig. 3b).

These two maps representing the spatial distributions of the energy potentials and integral intensities were derived from the C-mode measurement data. The luminescence intensity was higher at the bottom of the potential regions than in the surrounding hills, suggesting more frequent recombination of the excited carriers in the lower than in the higher potential regions of the semiconductor quantum well. The energy and intensity maps were consistently correlated with the results previously reported in Ref. 3.

### Two-probe measurement (normal: A→B)

**Fig. 4:**
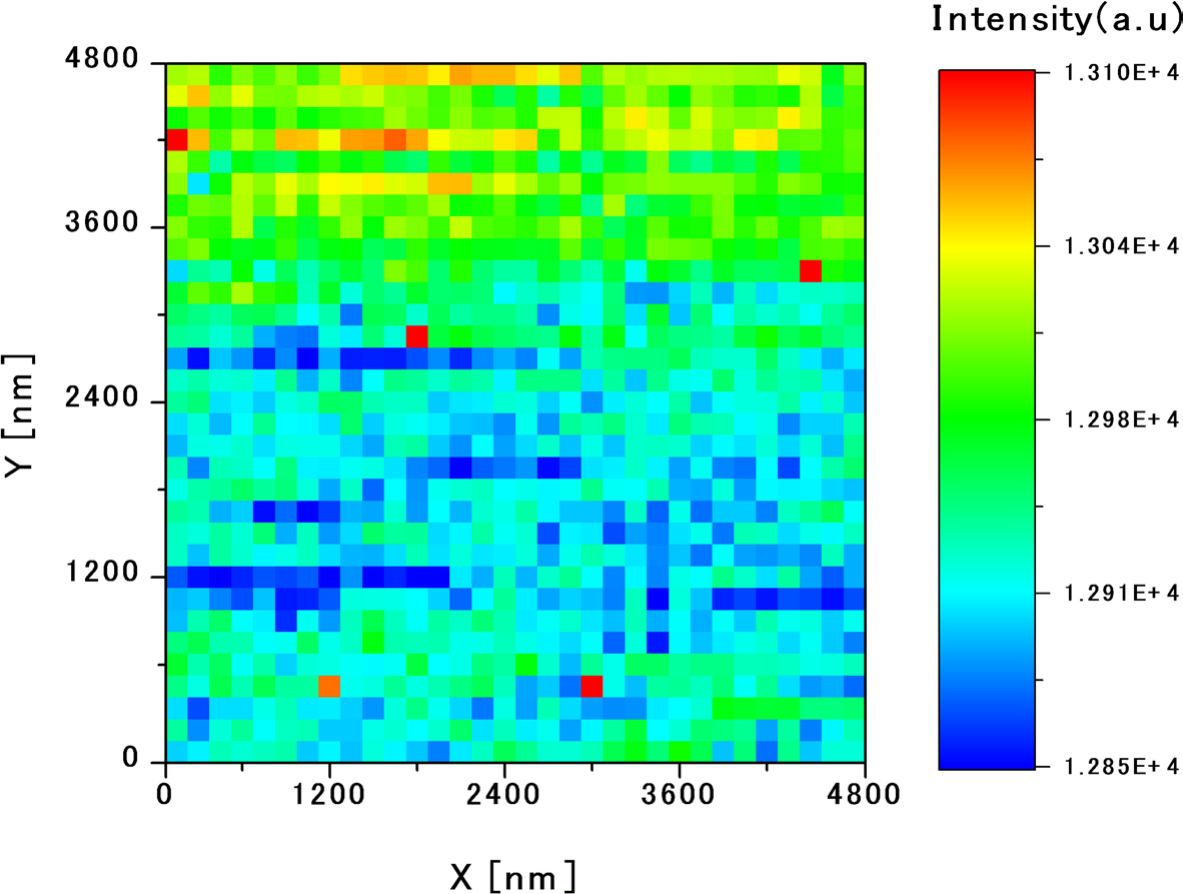
Integral intensity map based on the 2-probe measurements: excited locally at point A and detected by scanning using the S-probe and the C-probe, respectively. The excitation carriers generated at point A diffuse around the potential hill and recombine in the valleys between the hills, even in areas with relatively high energy.

Based on the energy and intensity distributions obtained from the C-mode measurements, a local excitation point, denoted as point A (filled upward triangle), was selected for 2-probe measurements. In this configuration, the S-probe locally excited the carriers at point A and the C-probe detected the PL resulting from recombination of the carriers diffusing from the excitation point. The C-probe detected the PL spectra over the area enclosed by the white dashed box (4.8 μm × 4.8 μm, 150-nm intervals, 1,024 points) in Fig. 3. Figure 4 shows the integrated intensity distribution map obtained from the 2-probe measurements.

Comparing the 2-probe intensity map (Fig. 4) with the C-mode energy-potential map (Fig. 3a), a higher intensity was observed in regions corresponding to lower potential energies, consistent with the previously observed C-mode intensity map. It appears that the carriers excited at point A reach and recombine in regions with relatively low-energy potentials.

However, the region near point A presents a relatively high-potential energy in the C-mode energy-potential map (Fig. 3a), suggesting that carriers must traverse energy barriers to reach low-energy areas. A more detailed analysis of Fig. 4 reveals a region of moderate luminescence intensity intersecting with a low-intensity region in the lower portion of the map. This region corresponds to an intermediate potential energy between the energy hills in Fig. 3. We suggest that excited carriers diffuse through the narrow paths of intermediate energy or bypass high-potential hills to reach the low-potential sites where recombination is more probable.

### Two-probe measurement (reversed: B→A)

To further investigate the carrier-transport dynamics, we swapped the roles of the excitation and detection probes for reversed 2-probe measurements. Based on the energy landscape obtained from the C-mode measurements, a new excitation point B (filled circle) was selected along with the previously used point A (filled upward triangle). In this configuration, the C-probe excited the carriers at point B and the S-probe observed the carrier recombination at point A. Note that this configuration reverses the previous configuration with excitation at point A and detection at point B. In this reversed arrangement, we could evaluate how the potential landscape of the sample influences the full carrier transport pathway encompassing the excitation, diffusion, and recombination processes.

**Fig. 5:**
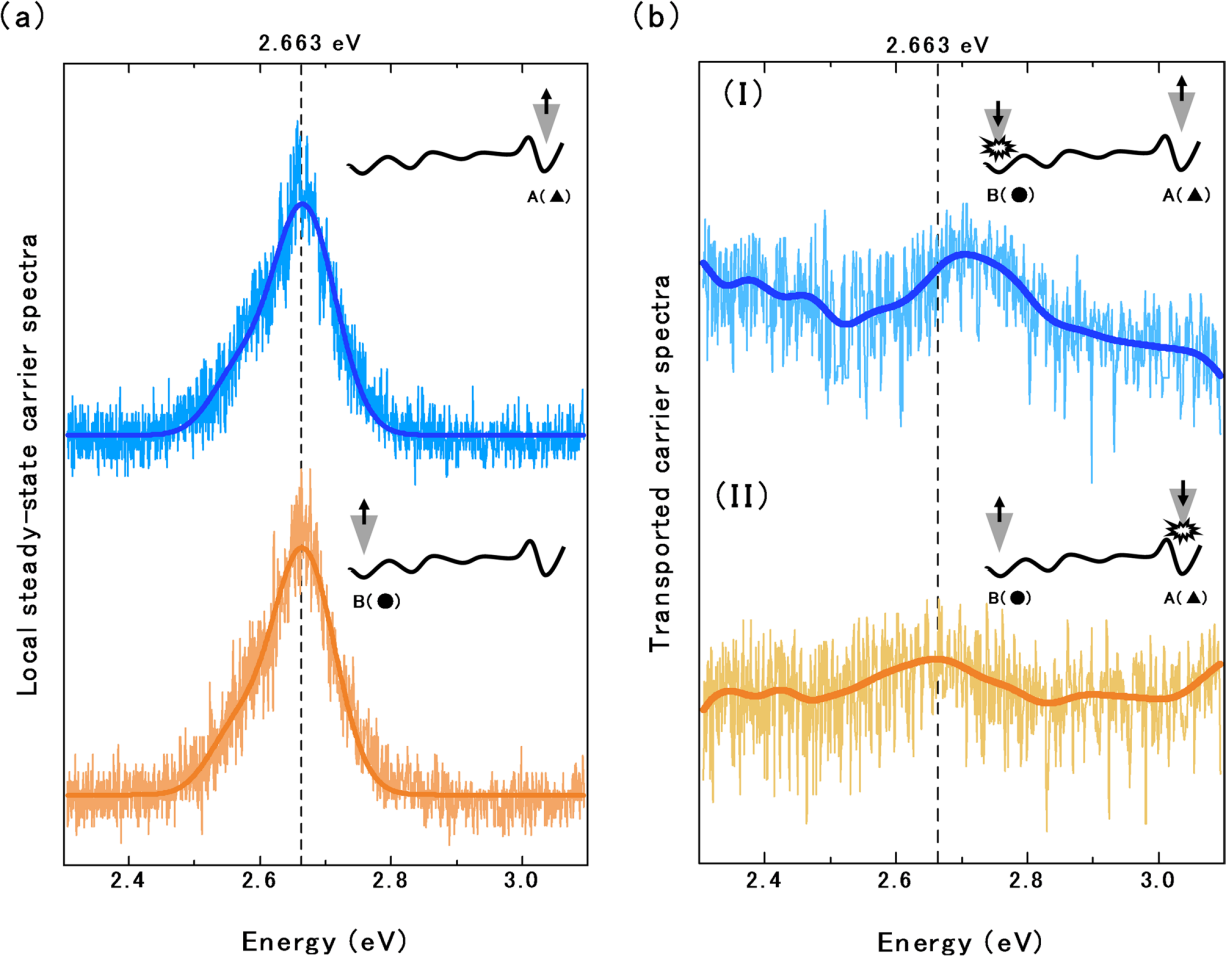
(a) Spectra detected at points A and B in C-mode and their fitting curves (thick lines). Both spectra show a central energy of 2.663 eV corresponding to the maximum intensity of the emission spectrum. (b) Spectra detected by 2-probe measurements and their moving averages (solid lines). Spectrum (I) was detected at point A using the configuration shown in Fig. 2d, and spectrum (II) was detected at point B using the configuration shown in Fig. 2c. The inset schematics show the average energy-potential height between the two points and the situation of excitation or detection by probes.

To assess the spectral response at each point, the emission spectra were obtained from the C-mode measurements at points A and B and their corresponding Lorentzian fitting curves (Fig. 5a). The central energy in both spectra was 2.663 eV, which was near the centre of the measured energy range. Under ideal conditions with negligible environmental influences, the spectra should be similar at points A and B. However, the central energy of the spectra obtained in case (I), when excitation and detection were applied at points B and A respectively, slightly differed from that in case (II), when the roles of the probes were reversed (Fig. 5b). It should be noted that the apparent intensity increases on the low-energy side in case (I) and on the high-energy side in case (II) of Fig. 5b arise from residual background.

The insets of Fig. 5 schematically show the average energy-potential height between the two points and the situation of excitation and detection by probes. The excited carriers generated at point A followed a downhill path toward point B. When excitation occurs near the potential hill (e.g., at point A), the excited carriers are expected to acquire sufficient initial energy (momentum) to surmount the energy hill and should retain high energies after some energy loss during diffusion. In contrast, the excited carriers generated at point B must reach the opposite side of the potential hill without being trapped in the surrounding potential valleys to reach point A. When excitation occurs at locations with no surrounding potential hills (e.g., at point B), the carriers lose energy before reaching the distant hills. In such instances, the hills are difficult or impossible to climb and only the high-energy components of the excited carriers can reach point A. Therefore, the peak central energy should be higher in the spectrum obtained at point A than in the spectrum obtained at point B.

In addition, excited carriers that cannot surmount the energy-potential hills either recombine locally or continue to diffuse through the energy landscape. As discussed above, such carriers avoid the energy hills and diffuse along the small valleys between the hills. The diffusion paths are also thought to influence the local density of states observed at the detection site.

**Fig. 6:**
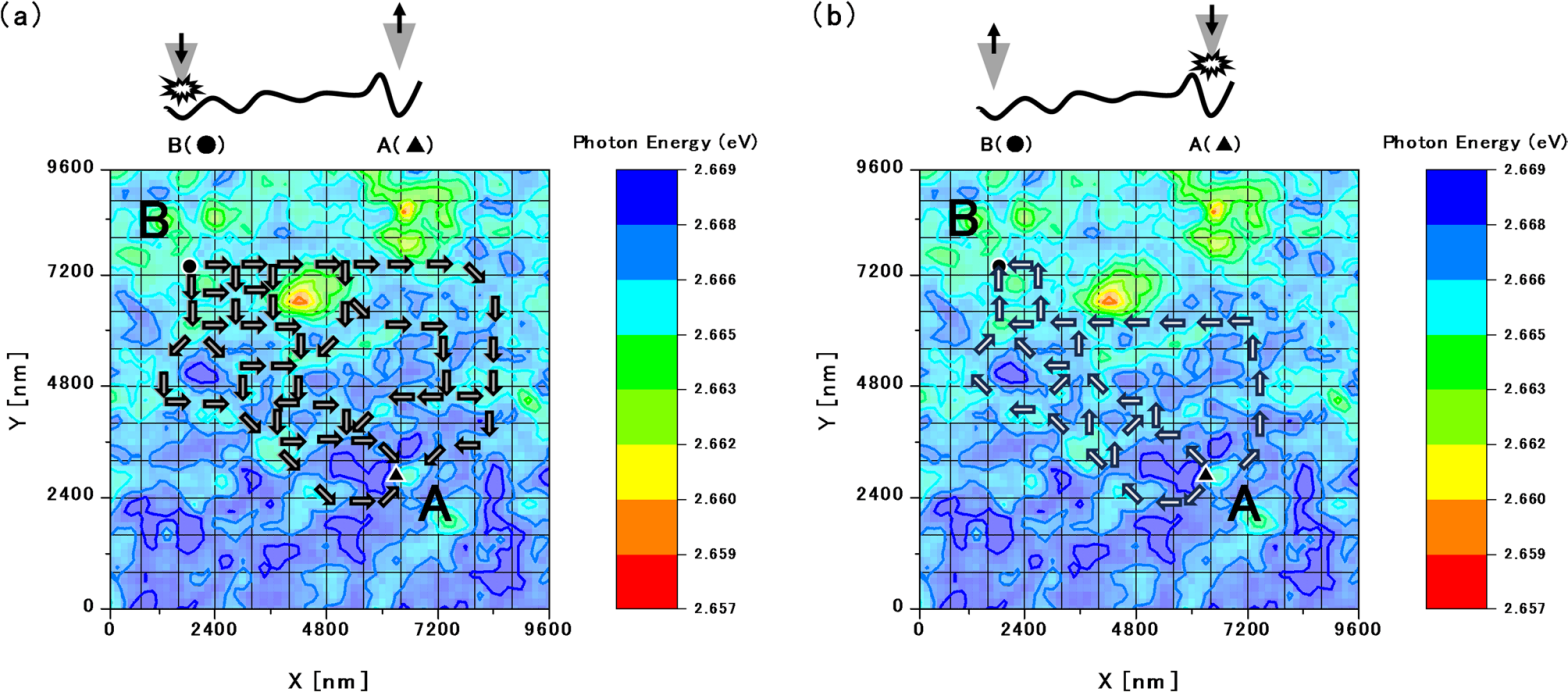
Diagrams predicting the possible diffusion paths of the excited carriers between point A and point B. Shown are the partial predictions of the diffusion paths followed by the excited carriers (a) generated at point B and moving toward A and (b) generated at point A. Multiple possible paths are predicted for the excited carriers generated at either point.

Figure 6 shows partial predictions of the possible paths chosen by the excited carriers diffusing between points A and B. The carriers excited at either point are expected to follow multiple diffusion paths. For example, the carriers generated at point B may reach point A by bypassing the potential energy hill and moving toward lower potential regions (Fig. 6a), whereas some carriers generated at point A that avoid immediate trapping in the potential valley may circumvent the energy hill to reach point B (Fig. 6b).

According to these results, multiple excited points at the same energy height on the potential surface exhibit different carrier dynamics that reflect their surrounding energy landscapes. Thus, the final state of the excited carriers is determined not only by the local conditions at the recombination sites but also by the complete transport process (i.e., generation, diffusion, and recombination).

Purely quantum transport should exhibit no directional differences because the probabilistic paths from B→A and A→B are symmetric. In addition, classical transport theory posits that carriers continuously lose energy and cannot overcome potential hills, diminishing the probability of excited carriers reaching distant detection points. However, during mesoscopic transport involving both quantum and classical properties, the higher-energy components of the excited carriers are expected to overcome potential barriers. This hypothesis must be confirmed through further investigations of quantum diffusion in this system. Additional experiments such as low-temperature measurements and detailed analysis would verify the quantum behaviour in the system. Nevertheless, the present results are sufficient to inspire theoretical studies in the mesoscopic region. In practice, at room temperature, thermal and quantum effects coexist at the mesoscopic scale, making their precise separation both experimentally and theoretically challenging. Our approach is characterised by measuring these inseparable effects collectively and basing our discussion on the resulting data. We respectfully note that, in the present discussion of the measurement results, the influence of V-defects^22^ and the quantum-confined Stark effect^23^ has not been treated separately. This is because the measurements were performed over a region larger than the scale at which such effects are typically significant, and thus, the results inherently comprehensively incorporate their influence. We acknowledge, however, that a more detailed separation of these effects would be valuable, and we consider this an important direction for future work. A detailed theoretical separation and interpretation remain challenges for future work.

## Summary

We generated and detected carriers and investigated their behaviour in a mesoscopic region. The objective was to infer the environmental effects on the excited carriers diffusing through the region. To this end, we locally detected the final-state density of locally excited carriers in a semiconductor multi-quantum well structure exhibiting nanoscale local structural changes within the intermediate scale region. Local spectroscopic measurements were performed using STM-assisted M-probe SNOM operated in C-mode and 2-probe mode.

Maps of the central emission energies and integrated emission intensities were constructed from the local spectroscopic spectra obtained in C-mode. Based on these results, we interpreted the spectra obtained in the 2-probe local spectroscopic measurements. The diffusion paths of the carriers excited by the local probe reflected the energy-potential terrain. We clarified that the carriers locally excited by the probe diffuse around regions with higher potential energy than the excitation-point energy. We exchanged the roles of the excitation and detection points to further discuss the behaviour of the diffusion carriers, revealing the bidirectional carrier transport.

After exchanging the starting point (excitation point) and end point (detection point) of 2-probe operation and comparing the resulting spectra, we identified differences between the final-state densities of excited carriers that had moved through the same region.

The final state of the excited carriers depends on the local conditions at the recombination point and the overall transport process, including generation, diffusion, and recombination. The generated carriers possess sufficient initial energy and gradually lose energy through diffusion. The observed differences cannot be explained by quantum transport alone, which predicts identical paths in both directions, or by classical transport alone, which predicts that carriers lose the energy required to overcome potential barriers and thus diffuse around peaks. However, in mesoscopic transport incorporating both quantum and classical properties, the energetically higher components are expected to overcome potential barriers. This prediction must be verified by investigating quantum diffusion in the mesoscopic region and the quantum behaviour must be verified in further experiments (e.g., low-temperature experiments) and analysis. Detailed theoretical separation and interpretation remain future work. The present measurements are expected to clarify the detailed behaviours of carriers in the intermediate regions of various materials, facilitating the development of new optoelectronic devices.

## Data Availability

Data sets generated during the current study are available from the corresponding author on reasonable request.
